# (*Z*)-*N*-{3-[(6-Chloro­pyridin-3-yl)meth­yl]-1,3-thia­zolidin-2-yl­idene}cyanamide

**DOI:** 10.1107/S1600536811013316

**Published:** 2011-04-16

**Authors:** Jin-Sheng Gao, Jun Qiao, Ying-Hui Yu, Guang-Feng Hou

**Affiliations:** a College of Chemistry and Materials Science, Heilongjiang University, Harbin 150080, People’s Republic of China; b Engineering Research Center of Pesticide of Heilongjiang Province, Heilongjiang University, Harbin 150080, People’s Republic of China

## Abstract

The asymmetric unit of the title compound, C_10_H_9_ClN_4_S, common name thia­cloprid, comprises two mol­ecules. In both mol­ecules, the thia­zolidine rings are almost planar (with r.m.s. deviations of 0.016 and 0.065 Å) and form dihedral angles of 73.36 (6) and 70.25 (8)° with the 2-chloro­pyridine rings. In the crystal, inter­molecular C—H⋯N hydrogen bonds links the mol­ecules into chains propagating in [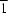
01].

## Related literature

For background to the title compound, a member of the neonicotinoide class of insecticides, see Maienfisch *et al.* (2003 ▶). For the synthesis, see Ishimitsu *et al.*, (1991 ▶)
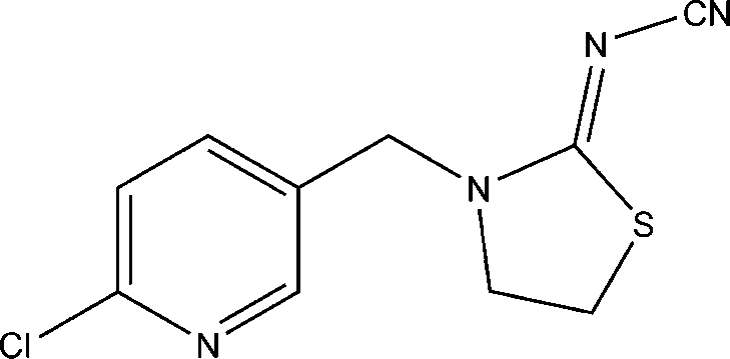


## Experimental

### 

#### Crystal data

C_10_H_9_ClN_4_S
*M* *_r_* = 252.73
Monoclinic, 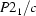

*a* = 7.1294 (14) Å
*b* = 35.469 (7) Å
*c* = 9.0211 (18) Å
β = 97.80 (3)°
*V* = 2260.1 (8) Å^3^
*Z* = 8
Mo *K*α radiation
μ = 0.50 mm^−1^
*T* = 293 K
0.31 × 0.29 × 0.20 mm

#### Data collection

Rigaku R-AXIS RAPID diffractometer
Absorption correction: multi-scan (*ABSCOR*; Higashi, 1995 ▶) *T*_min_ = 0.863, *T*_max_ = 0.909
21869 measured reflections
5151 independent reflections
3505 reflections with *I* > 2σ(*I*)
*R*_int_ = 0.046

#### Refinement

*R*[*F* ^2^ > 2σ(*F* ^2^)] = 0.048
*wR*(*F* ^2^) = 0.137
*S* = 1.08
5151 reflections
289 parameters
H-atom parameters constrained
Δρ_max_ = 0.21 e Å^−3^
Δρ_min_ = −0.29 e Å^−3^

### 

Data collection: *RAPID-AUTO* (Rigaku, 1998 ▶); cell refinement: *RAPID-AUTO*; data reduction: *CrystalClear* (Rigaku/MSC, 2002 ▶); program(s) used to solve structure: *SHELXS97* (Sheldrick, 2008 ▶); program(s) used to refine structure: *SHELXL97* (Sheldrick, 2008 ▶); molecular graphics: *SHELXTL* (Sheldrick, 2008 ▶); software used to prepare material for publication: *SHELXL97*.

## Supplementary Material

Crystal structure: contains datablocks I, global. DOI: 10.1107/S1600536811013316/kp2319sup1.cif

Structure factors: contains datablocks I. DOI: 10.1107/S1600536811013316/kp2319Isup2.hkl

Additional supplementary materials:  crystallographic information; 3D view; checkCIF report

## Figures and Tables

**Table 1:** Hydrogen-bond geometry (Å, °)

*D*—H⋯*A*	*D*—H	H⋯*A*	*D*⋯*A*	*D*—H⋯*A*
C3—H3⋯N5^i^	0.93	2.55	3.459 (4)	167
C13—H13⋯N1^ii^	0.93	2.49	3.408 (4)	169

Symmetry codes: (i) 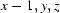; (ii) 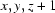.

**Fig. 1.**
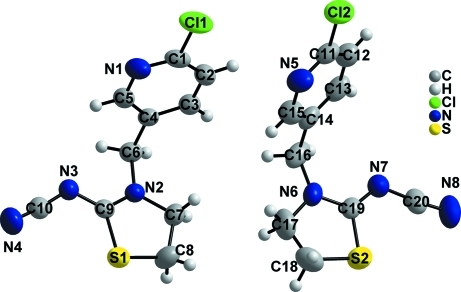
The molecular structure of the title compound showing displacement ellipsoids at the 50% probability level for non-H atoms.

**Fig. 2.**
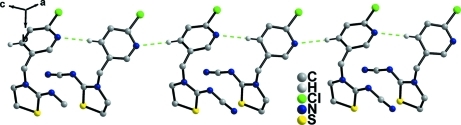
A partial packing view, showing the hydrogen bonding chain. Dashed lines indicate the hydrogen bonds, no involving H atoms have been omitted for clarity.

## supplementary crystallographic information

### Comment

Thiacloprid is the common name of the title compound, which is neonicotinoide class of insecticide. High efficacy and flexible application methods make it well suited for modern integrated pest management programs in many cropping systems (Ishimitsu *et al.*, 1991; Maienfisch *et al.*, 2003). We report here the synthesis and crystal structure of thiacloprid.

The asymmetric unit comprises two molecules; the thiazolidine rings are almost planar, and form the dihedral angles with 2-chloropyridine rings of 73.36 (6) and 70.25 (8)°, respectively (Fig. 1).

In the crystal, the intermolecular C—H···N hydrogen bonds link the molecules to form a chain (Fig. 2, Table 1).

### Experimental

The title compound was synthesised according the reference (Ishimitsu *et al.*, 1991). A mixture of 2-cyanoiminothiazolidine (12.7 g, 0.1 mol), 2-chloro-5-pyridylmethyl-chloride (17.4 g, 0.1 mol), and K_2_CO_3_ (41.4 g, 0.3 mol) in 150 mL of DMF was heated to 323 K and kept stirring for 7 h. After filtered under reduced pressure, the DMF solution was distilled off. A total of 20.2 g (80.2%) title compound was obtained after the recrystallisation from ethyl acetate (15 mL). The suitable colourless block crystal was picked out for the single crystal *X*-ray diffaction measurement.

### Refinement

H atoms bound to C atoms were placed in calculated positions and treated as riding on their parent atoms, with C—H = 0.93 Å (aromatic); C—H = 0.97 Å (methylene), and with *U*_iso_(H) = 1.2*U*_eq_(C).

### Crystal data

**Table:**

C_10_H_9_ClN_4_S	*F*(000) = 1040
*M**_r_* = 252.73	*D*_x_ = 1.486 Mg m^−^^3^
Monoclinic, *P*2_1_/*c*	Mo *K*α radiation, λ = 0.71073 Å
Hall symbol: -P 2ybc	Cell parameters from 12991 reflections
*a* = 7.1294 (14) Å	θ = 3.1–27.5°
*b* = 35.469 (7) Å	µ = 0.50 mm^−^^1^
*c* = 9.0211 (18) Å	*T* = 293 K
β = 97.80 (3)°	Block, colourless
*V* = 2260.1 (8) Å^3^	0.31 × 0.29 × 0.20 mm
*Z* = 8	

### Data collection

**Table:**

Rigaku R-AXIS RAPID diffractometer	5151 independent reflections
Radiation source: fine-focus sealed tube	3505 reflections with *I* > 2σ(*I*)
graphite	*R*_int_ = 0.046
ω scans	θ_max_ = 27.5°, θ_min_ = 3.1°
Absorption correction: multi-scan (*ABSCOR*; Higashi, 1995)	*h* = −8→9
*T*_min_ = 0.863, *T*_max_ = 0.909	*k* = −45→45
21869 measured reflections	*l* = −11→11

### Refinement

**Table:**

Refinement on *F*^2^	Primary atom site location: structure-invariant direct methods
Least-squares matrix: full	Secondary atom site location: difference Fourier map
*R*[*F*^2^ > 2σ(*F*^2^)] = 0.048	Hydrogen site location: inferred from neighbouring sites
*wR*(*F*^2^) = 0.137	H-atom parameters constrained
*S* = 1.08	*w* = 1/[σ^2^(*F*_o_^2^) + (0.0595*P*)^2^ + 0.4995*P*] where *P* = (*F*_o_^2^ + 2*F*_c_^2^)/3
5151 reflections	(Δ/σ)_max_ < 0.001
289 parameters	Δρ_max_ = 0.21 e Å^−^^3^
0 restraints	Δρ_min_ = −0.29 e Å^−^^3^

### Special details

**Table:**

Geometry. All e.s.d.'s (except the e.s.d. in the dihedral angle between two l.s. planes) are estimated using the full covariance matrix. The cell e.s.d.'s are taken into account individually in the estimation of e.s.d.'s in distances, angles and torsion angles; correlations between e.s.d.'s in cell parameters are only used when they are defined by crystal symmetry. An approximate (isotropic) treatment of cell e.s.d.'s is used for estimating e.s.d.'s involving l.s. planes.
Refinement. Refinement of *F*^2^ against ALL reflections. The weighted *R*-factor *wR* and goodness of fit *S* are based on *F*^2^, conventional *R*-factors *R* are based on *F*, with *F* set to zero for negative *F*^2^. The threshold expression of *F*^2^ > σ(*F*^2^) is used only for calculating *R*-factors(gt) *etc*. and is not relevant to the choice of reflections for refinement. *R*-factors based on *F*^2^ are statistically about twice as large as those based on *F*, and *R*- factors based on ALL data will be even larger.

### Fractional atomic coordinates and isotropic or equivalent isotropic displacement parameters (Å^2^)

**Table:**

	*x*	*y*	*z*	*U*_iso_*/*U*_eq_	
C1	0.8652 (3)	0.04805 (6)	0.2994 (3)	0.0528 (6)	
C2	0.7778 (4)	0.05431 (7)	0.4241 (3)	0.0564 (6)	
H2	0.8315	0.0461	0.5183	0.068*	
C3	0.6087 (4)	0.07309 (7)	0.4038 (3)	0.0541 (6)	
H3	0.5443	0.0777	0.4850	0.065*	
C4	0.5334 (3)	0.08520 (5)	0.2624 (3)	0.0417 (5)	
C5	0.6313 (4)	0.07628 (6)	0.1459 (3)	0.0525 (6)	
H5	0.5795	0.0835	0.0500	0.063*	
C6	0.3545 (3)	0.10860 (6)	0.2362 (3)	0.0486 (5)	
H6A	0.2856	0.1025	0.1390	0.058*	
H6B	0.2744	0.1024	0.3115	0.058*	
C7	0.4452 (4)	0.16785 (7)	0.3852 (3)	0.0586 (6)	
H7A	0.5677	0.1592	0.4331	0.070*	
H7B	0.3516	0.1622	0.4507	0.070*	
C8	0.4506 (6)	0.20899 (8)	0.3572 (4)	0.0831 (10)	
H8A	0.5573	0.2202	0.4200	0.100*	
H8B	0.3354	0.2207	0.3811	0.100*	
C9	0.4189 (3)	0.16902 (6)	0.1222 (3)	0.0422 (5)	
C10	0.4177 (3)	0.17589 (7)	−0.1287 (3)	0.0545 (6)	
C11	1.3337 (3)	0.05498 (7)	0.7709 (3)	0.0558 (6)	
C12	1.2138 (4)	0.04665 (8)	0.8736 (3)	0.0618 (6)	
H12	1.2387	0.0268	0.9406	0.074*	
C13	1.0549 (4)	0.06898 (8)	0.8737 (3)	0.0609 (7)	
H13	0.9712	0.0646	0.9423	0.073*	
C14	1.0214 (3)	0.09783 (7)	0.7712 (3)	0.0523 (6)	
C15	1.1509 (4)	0.10300 (8)	0.6735 (3)	0.0665 (7)	
H15	1.1280	0.1222	0.6033	0.080*	
C16	0.8483 (3)	0.12283 (9)	0.7672 (4)	0.0678 (8)	
H16A	0.7818	0.1234	0.6660	0.081*	
H16B	0.7636	0.1121	0.8315	0.081*	
C17	0.8939 (5)	0.19245 (10)	0.7102 (4)	0.0840 (10)	
H17A	0.7664	0.1963	0.6595	0.101*	
H17B	0.9753	0.1866	0.6353	0.101*	
C18	0.9605 (6)	0.22673 (9)	0.7904 (4)	0.0853 (10)	
H18A	0.8799	0.2478	0.7556	0.102*	
H18B	1.0887	0.2325	0.7728	0.102*	
C19	0.9155 (3)	0.17075 (7)	0.9595 (3)	0.0464 (5)	
C20	0.9267 (3)	0.15741 (8)	1.2059 (3)	0.0593 (6)	
Cl1	1.08715 (10)	0.02601 (2)	0.31970 (11)	0.0811 (3)	
Cl2	1.53924 (11)	0.02813 (2)	0.76846 (10)	0.0809 (2)	
N1	0.7967 (3)	0.05776 (6)	0.1620 (3)	0.0592 (5)	
N2	0.3963 (2)	0.14899 (5)	0.2425 (2)	0.0432 (4)	
N3	0.4000 (3)	0.15412 (5)	−0.0121 (2)	0.0504 (5)	
N4	0.4288 (4)	0.19221 (7)	−0.2375 (3)	0.0777 (7)	
N5	1.3080 (3)	0.08234 (7)	0.6720 (3)	0.0658 (6)	
N6	0.8957 (3)	0.16118 (6)	0.8155 (2)	0.0540 (5)	
N7	0.9051 (3)	0.14604 (6)	1.0652 (2)	0.0551 (5)	
N8	0.9439 (4)	0.16423 (9)	1.3319 (3)	0.0864 (8)	
S1	0.47264 (10)	0.216369 (16)	0.16494 (8)	0.05802 (19)	
S2	0.95422 (12)	0.21907 (2)	0.98475 (9)	0.0698 (2)	

### Atomic displacement parameters (Å^2^)

**Table:**

	*U*^11^	*U*^22^	*U*^33^	*U*^12^	*U*^13^	*U*^23^
C1	0.0496 (12)	0.0361 (10)	0.0750 (18)	0.0031 (10)	0.0159 (12)	0.0066 (11)
C2	0.0653 (14)	0.0536 (13)	0.0499 (15)	0.0087 (12)	0.0069 (12)	0.0066 (11)
C3	0.0656 (14)	0.0505 (12)	0.0483 (14)	0.0063 (11)	0.0159 (11)	0.0021 (11)
C4	0.0476 (11)	0.0336 (9)	0.0458 (13)	−0.0015 (9)	0.0138 (9)	0.0020 (8)
C5	0.0664 (14)	0.0486 (12)	0.0450 (14)	0.0081 (11)	0.0160 (11)	0.0053 (10)
C6	0.0465 (11)	0.0424 (11)	0.0580 (15)	−0.0045 (9)	0.0113 (10)	0.0002 (10)
C7	0.0753 (16)	0.0539 (13)	0.0462 (14)	−0.0019 (12)	0.0061 (12)	−0.0049 (11)
C8	0.133 (3)	0.0574 (16)	0.060 (2)	0.0008 (18)	0.0185 (19)	−0.0117 (14)
C9	0.0367 (10)	0.0416 (10)	0.0476 (13)	0.0033 (8)	0.0029 (9)	0.0007 (9)
C10	0.0555 (13)	0.0566 (13)	0.0501 (15)	0.0046 (11)	0.0032 (11)	0.0001 (12)
C11	0.0568 (13)	0.0556 (13)	0.0579 (16)	−0.0027 (11)	0.0177 (12)	−0.0152 (12)
C12	0.0708 (16)	0.0605 (15)	0.0575 (17)	−0.0074 (13)	0.0214 (13)	−0.0025 (12)
C13	0.0601 (14)	0.0736 (16)	0.0541 (16)	−0.0178 (13)	0.0257 (12)	−0.0177 (13)
C14	0.0495 (12)	0.0619 (14)	0.0465 (14)	−0.0090 (11)	0.0106 (10)	−0.0205 (11)
C15	0.0728 (17)	0.0727 (17)	0.0591 (18)	0.0088 (14)	0.0279 (14)	0.0006 (13)
C16	0.0482 (13)	0.0861 (19)	0.0685 (19)	−0.0033 (13)	0.0062 (12)	−0.0297 (15)
C17	0.096 (2)	0.112 (3)	0.0459 (17)	0.009 (2)	0.0141 (15)	0.0145 (17)
C18	0.106 (2)	0.0733 (19)	0.080 (2)	0.0222 (18)	0.0239 (19)	0.0220 (17)
C19	0.0389 (10)	0.0567 (13)	0.0438 (13)	0.0051 (10)	0.0068 (9)	−0.0056 (10)
C20	0.0484 (12)	0.0786 (17)	0.0525 (16)	0.0114 (12)	0.0125 (11)	0.0098 (13)
Cl1	0.0568 (4)	0.0582 (4)	0.1311 (8)	0.0141 (3)	0.0232 (4)	0.0113 (4)
Cl2	0.0785 (5)	0.0756 (5)	0.0938 (6)	0.0180 (4)	0.0307 (4)	−0.0044 (4)
N1	0.0684 (13)	0.0531 (11)	0.0623 (15)	0.0100 (10)	0.0310 (11)	0.0084 (10)
N2	0.0454 (9)	0.0407 (9)	0.0437 (11)	0.0020 (8)	0.0068 (8)	−0.0008 (8)
N3	0.0559 (11)	0.0496 (10)	0.0450 (12)	0.0029 (9)	0.0045 (9)	−0.0002 (9)
N4	0.0968 (18)	0.0831 (17)	0.0537 (15)	0.0063 (14)	0.0119 (13)	0.0126 (13)
N5	0.0701 (14)	0.0680 (13)	0.0662 (15)	0.0064 (11)	0.0349 (12)	−0.0018 (11)
N6	0.0504 (10)	0.0702 (13)	0.0413 (11)	0.0095 (10)	0.0058 (8)	−0.0082 (9)
N7	0.0581 (11)	0.0601 (12)	0.0485 (13)	0.0040 (10)	0.0129 (9)	0.0025 (9)
N8	0.0813 (17)	0.133 (2)	0.0470 (15)	0.0174 (16)	0.0147 (12)	0.0044 (15)
S1	0.0681 (4)	0.0420 (3)	0.0615 (4)	−0.0052 (3)	−0.0003 (3)	0.0019 (3)
S2	0.0892 (5)	0.0566 (4)	0.0649 (5)	0.0004 (3)	0.0145 (4)	−0.0053 (3)

### Geometric parameters (Å, °)

**Table:**

C1—N1	1.314 (3)	C11—N5	1.314 (4)
C1—C2	1.376 (4)	C11—C12	1.375 (4)
C1—Cl1	1.752 (2)	C11—Cl2	1.750 (3)
C2—C3	1.368 (3)	C12—C13	1.383 (4)
C2—H2	0.9300	C12—H12	0.9300
C3—C4	1.383 (3)	C13—C14	1.378 (4)
C3—H3	0.9300	C13—H13	0.9300
C4—C5	1.375 (3)	C14—C15	1.373 (4)
C4—C6	1.513 (3)	C14—C16	1.516 (4)
C5—N1	1.340 (3)	C15—N5	1.340 (4)
C5—H5	0.9300	C15—H15	0.9300
C6—N2	1.463 (3)	C16—N6	1.454 (3)
C6—H6A	0.9700	C16—H16A	0.9700
C6—H6B	0.9700	C16—H16B	0.9700
C7—N2	1.451 (3)	C17—N6	1.459 (4)
C7—C8	1.482 (4)	C17—C18	1.461 (5)
C7—H7A	0.9700	C17—H17A	0.9700
C7—H7B	0.9700	C17—H17B	0.9700
C8—S1	1.782 (3)	C18—S2	1.781 (4)
C8—H8A	0.9700	C18—H18A	0.9700
C8—H8B	0.9700	C18—H18B	0.9700
C9—N3	1.312 (3)	C19—N7	1.305 (3)
C9—N2	1.325 (3)	C19—N6	1.331 (3)
C9—S1	1.754 (2)	C19—S2	1.746 (2)
C10—N4	1.152 (3)	C20—N8	1.152 (4)
C10—N3	1.324 (3)	C20—N7	1.321 (3)
			
N1—C1—C2	125.2 (2)	C14—C13—C12	119.5 (2)
N1—C1—Cl1	115.5 (2)	C14—C13—H13	120.2
C2—C1—Cl1	119.3 (2)	C12—C13—H13	120.2
C3—C2—C1	117.2 (2)	C15—C14—C13	117.3 (2)
C3—C2—H2	121.4	C15—C14—C16	121.5 (3)
C1—C2—H2	121.4	C13—C14—C16	121.1 (3)
C2—C3—C4	120.0 (2)	N5—C15—C14	124.7 (3)
C2—C3—H3	120.0	N5—C15—H15	117.6
C4—C3—H3	120.0	C14—C15—H15	117.6
C5—C4—C3	117.4 (2)	N6—C16—C14	112.62 (19)
C5—C4—C6	120.7 (2)	N6—C16—H16A	109.1
C3—C4—C6	121.9 (2)	C14—C16—H16A	109.1
N1—C5—C4	124.1 (2)	N6—C16—H16B	109.1
N1—C5—H5	118.0	C14—C16—H16B	109.1
C4—C5—H5	118.0	H16A—C16—H16B	107.8
N2—C6—C4	111.60 (17)	N6—C17—C18	109.7 (3)
N2—C6—H6A	109.3	N6—C17—H17A	109.7
C4—C6—H6A	109.3	C18—C17—H17A	109.7
N2—C6—H6B	109.3	N6—C17—H17B	109.7
C4—C6—H6B	109.3	C18—C17—H17B	109.7
H6A—C6—H6B	108.0	H17A—C17—H17B	108.2
N2—C7—C8	108.2 (2)	C17—C18—S2	108.1 (2)
N2—C7—H7A	110.1	C17—C18—H18A	110.1
C8—C7—H7A	110.1	S2—C18—H18A	110.1
N2—C7—H7B	110.1	C17—C18—H18B	110.1
C8—C7—H7B	110.1	S2—C18—H18B	110.1
H7A—C7—H7B	108.4	H18A—C18—H18B	108.4
C7—C8—S1	108.5 (2)	N7—C19—N6	122.2 (2)
C7—C8—H8A	110.0	N7—C19—S2	125.99 (19)
S1—C8—H8A	110.0	N6—C19—S2	111.81 (18)
C7—C8—H8B	110.0	N8—C20—N7	174.3 (3)
S1—C8—H8B	110.0	C1—N1—C5	116.1 (2)
H8A—C8—H8B	108.4	C9—N2—C7	116.01 (19)
N3—C9—N2	122.1 (2)	C9—N2—C6	122.68 (19)
N3—C9—S1	125.52 (18)	C7—N2—C6	120.61 (19)
N2—C9—S1	112.34 (17)	C9—N3—C10	119.3 (2)
N4—C10—N3	174.2 (3)	C11—N5—C15	115.9 (2)
N5—C11—C12	125.0 (2)	C19—N6—C16	121.3 (2)
N5—C11—Cl2	115.7 (2)	C19—N6—C17	115.6 (2)
C12—C11—Cl2	119.3 (2)	C16—N6—C17	122.3 (2)
C11—C12—C13	117.5 (3)	C19—N7—C20	119.1 (2)
C11—C12—H12	121.3	C9—S1—C8	91.61 (12)
C13—C12—H12	121.3	C19—S2—C18	92.62 (14)

### Hydrogen-bond geometry (Å, °)

**Table:**

*D*—H···*A*	*D*—H	H···*A*	*D*···*A*	*D*—H···*A*
C3—H3···N5^i^	0.93	2.55	3.459 (4)	167
C13—H13···N1^ii^	0.93	2.49	3.408 (4)	169

Symmetry codes: (i) *x*−1, *y*, *z*; (ii) *x*, *y*, *z*+1.
